# Young Men’s Communication Needs for the Human Papillomavirus (HPV) Vaccine: A Cross-Cultural, Qualitative Analysis in Scotland, Spain, and the USA

**DOI:** 10.1007/s12529-025-10387-6

**Published:** 2025-08-07

**Authors:** Carol Gray Brunton, Elaine Carnegie, Janette Pow, Irina Todorova, Dafina Petrova, Rocio Garcia-Retamero, Anne Whittaker

**Affiliations:** 1 School of Health and Social Care, Edinburgh Napier University, Edinburgh, EH11 4BN UK; 2 Department of Applied Psychology, Bouvé College of Health Sciences, Northeastern University, Boston, USA; 3 Investigación Biosanitaria ibs.Granada, Granada, Spain, Escuela Andaluza de Salud Pública, Granada, Spain; 4 CIBER of Epidemiology and Public Health (CIBERESP), Madrid, Spain; 5 Virgen de las Nieves University Hospital, Granada, Spain; 6 Department of Experimental Psychology, University of Granada, Granada, Spain; 7 Faculty of Health Sciences & Sport, Pathfoot Building, University of Stirling, Stirling, FK9 4LA UK

**Keywords:** HPV vaccine, Vaccine hesitancy, Young men, Communication, Qualitative, Cross-cultural

## Abstract

**Background:**

Globally, human papillomavirus (HPV) vaccine uptake rates have declined within a wider context of vaccine hesitancy. Gender-neutral vaccine programmes are increasing and recommended for the prevention of cervical, anogenital, and oropharyngeal cancers affecting both women and men. To address the problem of suboptimal HPV uptake rates for boys and men, we aimed to understand young men’s communication needs for HPV messages in a cross-cultural analysis.

**Methods:**

Qualitative methods consisting of 14 focus group discussions were conducted with young men (18–26 years) about the HPV vaccine in three countries with varying HPV vaccine implementation policies at the time. A total of 55 participants were included from Scotland (*n*=14), Spain (*n*=25), single-gendered programmes, and the USA (*n*=16), gender-neutral programme. Analysis was informed by reflexive thematic analysis.

**Findings:**

Four themes were identified: (1) the ‘*Girl* vaccine’: primarily seen as a vaccine for girls, despite varying vaccine knowledge and awareness across contexts; (2) vaccine a*mbivalence*: perceptions of men’s HPV risks were low and some vaccine worries were evident from US contexts; (3) *altruism*: a pervasive theme related to young men’s roles and responsibilities for HPV transmission within relationships; (4) *trusted sources:* messages from health care providers/authorities were persistently valued but direct opportunities were often absent.

**Conclusion:**

Public health messaging to increase HPV vaccination for young men should focus on *direct* appeal to young men’s health through specific and targeted HPV messages for cancer and sexual health benefits to increase the personal relevance of the vaccine. It should also focus on *indirect appeal* through incorporating messages about safe sex in relationships and for herd immunity. Healthcare providers and authorities are trusted sources of information and can influence men’s confidence for the HPV vaccine; opportunities for interactions should be optimised.

## Introduction

Vaccine hesitancy is defined as a delay or refusal to accept vaccines despite vaccine availability and is a top ten threat to public health [1]. Vaccine hesitancy is complex and context-specific depending on confidence, complacency, and convenience [1]. Uptake rates for one vaccine, the human papillomavirus vaccine (HPV), have declined by 15% since 2019 and the global COVID-19 pandemic— the greatest decline in uptake rates compared with other vaccines [2]. The HPV vaccine provides protection against common strands of the sexually transmitted HPV virus, which is the most common sexually transmitted infection amongst young adults [3]. HPV infection was attributed to 690,000 new cases of cancer per year in 2018 [4]. Persistent infections with oncogenic HPV types are estimated to cause 90% of cervical and anal cancers, 70% of vulvar and vaginal cancers, 60% of penile cancer, and 70% of oropharyngeal cancers, and low-risk HPV infections are associated with genital warts [3, 4]. Preadolescent girls have been primarily targeted with the HPV vaccine prior to sexual debut to reduce the incidence and burden of cervical cancer worldwide, and the vaccine has been shown to be highly successful as a cancer-prevention vaccine [3, 5]. For example, there were no new incidences of invasive cases of cervical cancer for the cohort of vaccinated females in Scotland’s national HPV programme [6]. Licensed since 2006, national vaccine policies are continually changing, and there are now six HPV vaccines with 125 countries implementing national vaccine programmes (single-gendered) and 47 countries currently offering provision for boys (gender-neutral) [2, 3, 5]. Vaccinating boys is recommended if supplies are available in some resource-scarce contexts, but vaccinating girls is the priority [4, 7].

Despite the success of the cancer-preventing HPV vaccine for cervical cancer in women in some contexts, there is also increasing and compelling evidence for the health benefits of the HPV vaccine for boys/young men, which has been overlooked for cancers affecting the penis, anus, head and neck, and genital warts [8–11]. A recent systematic review and meta-analysis highlighted that the global prevalence of high-risk genital HPV rates was high across the lifespan in a general population of heterosexual men and that this was consistent with the evidence showing men were a ‘reservoir’ for HPV general infection [12].

Europe’s Cancer Plan recommends equitable, gender-neutral vaccine programmes to foster ‘herd’ or population immunity and to ensure that 90% of girls are vaccinated across the region by 2030 and that vaccination for boys is significantly increased [13]. Although vaccine hesitancy appears to be a global phenomenon, European contexts appear to be particularly affected [13, 14]. Vaccine confidence in European contexts depended on the context and population according to a systematic review which included the quantity and quality of information sources for the HPV vaccine, worries about side effects, mistrust of health authorities/workers, and new vaccines [14]. Additionally, parents’ uptake of HPV vaccines for their children showed suboptimal levels amongst boys; this was twofold compared with girls, in a systematic review and meta-analysis from 15 countries [15]. Physician recommendation of the vaccine for both boys and girls, addressing parental concerns about vaccines, and promoting positive beliefs about vaccines as well as cost reimbursements were recommended to increase uptake rates amongst boys/young men [15]. Surveys in the United Kingdom (UK) measured parents’ willingness to have their teenage sons vaccinated with the HPV vaccine, following the UK’s Joint Committee for Vaccination and Immunisation (JCVI) recommendation to include boys in the national immunisation programme in 2019 [16, 17]. Results showed poor parental knowledge of HPV, particularly in relation to its impact on boys’ health compared with girls, but most parents reported being willing to have their sons vaccinated after receiving some basic information about the HPV vaccine [16, 17]. The main barriers towards the uptake of HPV vaccination for boys/young men from several contexts in a narrative review indicated a lack of knowledge, general vaccine hesitancy, lack of recommendation or discussion with a healthcare professional, cost and logistical factors, and ideas about promiscuity [18].

HPV vaccine uptake rates amongst boys/young men are suboptimal, despite now being offered in many countries that have moved from single-gendered vaccine campaigns to gender-neutral vaccine campaigns [15]. In Scotland, for example, boys were persistently less likely than girls to be vaccinated with the HPV vaccine, with a persistent and growing gap: 2022/2023 statistics showed an overall 76.3% uptake for girls and 69.7% for boys for the first dose [19]. In the United States (US), girls were more likely to be vaccinated with at least one dose of the HPV vaccine (42.9%) compared with boys (34.6%) [20] and young males were most likely to remain unvaccinated in the US, despite having attended a physician visit [21]. Changes to national vaccine policy to vaccinate boys in Scotland in 2019 and in Spain in 2021 are in keeping with current recommendations [7]. However, such implementation coincided with the COVID-19 pandemic and thus will have impacted HPV vaccination implementation efforts for boys. At the same time, the WHO’s Strategic Advisory Group of Experts on Immunization (SAGE) recommended the temporary suspension of vaccination of boys until girls were reached due to vaccine shortages [22]. Hence, vaccine implementation for boys/young men has been slower to start, and messages about boys’ vaccination have been mixed. Vaccine hesitancy was particularly salient during COVID-19 [2, 7].

HPV vaccination in boys/young men is important to ensure ‘herd’ immunity and reach targets of 90% uptake rates for women and increase uptake amongst boys/young men [13] for prevention of not only cervical cancer but also anogenital and oropharyngeal cancers affecting both women and men [8–11]. To understand the problem of persisting suboptimal HPV vaccine uptake rates amongst boys/young men and address this, it is important to include men’s views on communication for HPV information so that improved communication approaches can be adopted to reach more boys/young men in vaccination programmes. Qualitative research approaches have the potential to offer rich depth and understandings in vaccination research in specific contexts [23]. Sledge et al. [24] considered the male voice for young men’s communication preferences about HPV in a qualitative sample in the US (the Midwest). They found that there was no preferred ‘one size fits all’ approach and that health care providers should be trained and aware in cancer prevention for men in that context. Qualitative, cross-cultural understanding can shed unique insights into different cultural contexts and young men’s communication needs for the HPV vaccine where this remains a problem. We can draw insights from different cultural contexts with differing vaccine policies (i.e. single versus moving to a gender-neutral vaccine programme) as vaccine policy is continually changing. There is an absence of the male voice from different cultural contexts with most deriving from North American contexts and quantitative results do not explain the reasons for differences in uptake rates. In depth, qualitative cross-cultural understandings can inform communication strategies in different contexts and share learnings and insights where HPV vaccine policies differ or change. Our aim was to provide rich, in-depth understandings of young men’s views and communication needs for HPV through accounts produced in different cultural contexts including Scotland, Spain, and the United States. These represented single-gendered and gender-neutral vaccine programmes at the time.

## Method

### Participants

We recruited young men from Scotland, Spain, and the United States for this cross-cultural qualitative study about the HPV vaccine in a purposive sample. The study was advertised via e-mailing lists, posters, or in-class announcements at local universities and higher education colleges in urban areas. The only criterion for inclusion was that men be between 18 and 26 years old. This age group was selected for pragmatic reasons as young men could consent to participate in research. There were other reasons to include this age group as they have higher risks of HPV due to increased sexual activity [12]. It was emphasised that knowledge about HPV or vaccination history was not required for study participation. Participants were reimbursed for their time with small shop vouchers (equivalent of £10) or course credit as participation in their courses as per good practice guidance for research participants [25]. Data were originally collected in 2015. At this time, only the US offered the HPV vaccine to boys/young men in private health provision. Scotland and Spain did not routinely offer the HPV vaccine to boys and adopted single-gendered vaccine programmes including national school-based provision (Scotland) and on-demand regional provision through health centres in Andalusia (Spain). The HPV vaccine was extended to boys in Scotland in 2019 and in Spain in 2021 in gender-neutral vaccine campaigns [7]. However, the COVID-19 pandemic saw global HPV vaccine rates decline and affected the implementation of the HPV vaccine for boys [2]. WHO recommendations to temporarily suspend vaccination for boys globally may have affected the messaging of this vaccine for boys/men [22]. There is value in considering our data from a cross-cultural perspective, given persistent efforts to promote HPV vaccine uptake rates amongst boys/young men since the adoption of gender-neutral vaccine programmes and the history around HPV vaccination implementation for boys/young men.

## Ethics

All procedures performed in studies involving human participants were in accordance with the ethical standards of the universities (through the primary institution Edinburgh Napier University Project Number 296117, and subsequently University of Granada, Northeastern University) and with the 1964 Helsinki declaration and its later amendments or comparable ethical standards. Informed consent principles were applied in this study whereby voluntary participation was emphasised; participants were informed that they could withdraw from the study at any point, and potential risks and benefits of taking part were discussed. Participants could refuse to answer any of the focus group questions and did not need to have any specific knowledge about the topic. Information both verbal and written was given to the participants prior to signing a consent form. We paid attention to the gender composition of the focus group facilitators and attempted to ensure that there were young men facilitators in most of the focus groups. This we achieved in the Scottish and Spanish focus groups, but we were not able to achieve this in the US discussions. All personal identifiable information was removed from the data and participant names were replaced with ID codes.

## Data Collection

Fourteen focus group discussions were conducted with a total of 55 participants; these are appropriate for collecting data about people’s views or negotiating controversial topics [26]. Five focus groups were conducted in Scotland (*n*=14), three in Spain (*n*=25), and six in the US (*n*=16). Participants were mostly university or higher education students. Here, and in the analysis section, we outline our steps to enhance rigour and transparency in the study, which were mainly through following the same data collection procedures in all countries and recording and transcribing discussions; briefing local researchers in all countries; detailing a systematic process to coding and thematic development; using illustrative data quotes to enhance the transparency of our analysis; labelling quotes and context; and paying attention to ‘negative’ cases [27]. Prior to the discussions and following informed consent procedures described above, participants filled in a short survey covering demographic data and questions related to HPV. The same topic guide was followed in all countries, and similar stimulus materials were distributed as prompts for the discussion (e.g. local information leaflets about the HPV vaccine specific to each country were provided, information for boys/young men provided by the US Centre for Disease Control (CDC) was also provided and any local news articles). Discussion topics included views of vaccination in general, knowledge or opinions regarding HPV and the HPV vaccine, relevance of the vaccine for oneself and others (e.g. girls and boys), and information and communication sources for the HPV vaccine. Local researchers, trained in qualitative research and supervised by experienced qualitative researchers, facilitated the focus group discussions, which lasted approximately one hour each. The same topic guides were used in all contexts (Table 1).

**Table 1:** Focus group topic guide

• Views and experiences of vaccination in general
• Knowledge/recall about the HPV virus and the HPV vaccine
• Targets of the HPV vaccine [Prompt: Show Local/ECCA information brochure at this stage for girls/young women; 5-10 mins]
• Relevance of the HPV vaccine for you [Prompt: Show CDC US brochure given at this stage; 5-10 mins]
• Information seeking for vaccination/more information [Prompt: approximately half way or 40 minutes into one hour discussion, present participants with media reports 2-3 in local country. Pass these around and let people read these 5-10 mins].
• Anything to add about the HPV vaccine or virus to the discussion
• Summary: one or two most important points from the discussion

## Analysis

In keeping with qualitative research, discussions were audio-recorded and transcribed verbatim. The Spanish transcripts were translated into English by speakers fluent in both languages, and random subsets were back translated and compared with the originals to ensure quality and enable analysis [28]. Previously, we outlined different qualitative methodological approaches and underlying assumptions for exploring HPV views and communication needs amongst young women, including Foucauldian discourse analysis (FDA) and thematic analysis [29–31]. Braun and Clarke’s reflexive thematic analysis was used as our overriding analytical approach [32, 33]. This was underpinned by a social constructionist lens [34]. This posits that participants are both enabled and constrained by their accounts and that it is important to understand these accounts to understand social ‘realities’ within a particular context. This was a particularly pragmatic choice given that we were working with cross-cultural data. It enabled us to consider the possibilities and the limits within the young men’s accounts and the wider contexts in which they occurred. Initial coding was conducted systematically for each transcript by at least two experienced coders and led by GCB. Codes were organised and grouped into themes, which were then named, discussed, and revised to build rigour and credibility and to explore nuances and differences [27, 32, 33]. Analysis was originally conducted separately by region and vaccine programme (Europe and US) and presented as part of a conference symposium [35]. We then undertook cross-cultural comparisons from young men’s discussions regarding communication needs for the HPV vaccine in single and gender-neutral vaccine programmes to shed insight into men’s communication needs, given this was a persistent, current problem. We conducted our cross-cultural analysis of the wider dataset to identify overarching and salient themes that were pervasive.

## Findings

### Descriptive Findings of Basic Demographic Characteristics and Prior HPV Knowledge and Experience  

From Table 1, prior to the focus group discussions, 14/16 (87.5%) of US participants reported that they had heard of the HPV virus, 13/16 (81%) had heard of the HPV vaccine, and 11/16 (68.7%) knew that the HPV virus was linked with cancer. In the US sample, 6/16 (38%) reported that they had been previously vaccinated with at least one dose of the HPV vaccine. In Scotland, 9/14 (64%) reported having previously heard of the HPV virus, 8/14 (57%) had heard of the HPV vaccine, and 7/14 (50%) reported knowing that the HPV virus was linked with cancer. No Scottish participants reported that they had been previously vaccinated with HPV. In the Spanish sample, 15/25 (60%) had heard of the HPV virus, 7/25 (28%) had heard of the HPV vaccine, and 11/25 (44%) knew that the HPV virus was linked with cancer. No Spanish participants reported that they had been previously vaccinated with HPV (Table 2).

**Table 2:** Basic demographic characteristics of participants and prior HPV knowledge and experience (*N*=55)

	**Scotland**	**Spain**	**US**
*N*	14	25	16
Mean age (±SD)	23 (±3)	20 (±2)	20 (±2)
Not sexually active	4 (29%)	3 (12%)	5 (31%)
Heard of HPV Virus	9 (64%)	15 (60%)	14 (87.5%)
Heard of HPV Vaccine	8 (57%)	7 (28%)	13 (81%)*
Know that HPV virus was linked with cancer	7 (50%)	11 (44%)	11 (68.7%)
Should boys/young men be vaccinated	Yes 6 (42.8%) Unsure 8 (57%) No 0 (0%)	Yes 5 (20%) Unsure 17(68%) No 3 (12%)	Yes 11(68.7%) Unsure 5 (31%) No 0 (0%)

^*^Six participants (38%) from the US were vaccinated with at least one dose of the HPV vaccine

## Themes

We identified four salient themes from our cross-cultural analysis: (1) the ‘Girl vaccine’; (2) *vaccine ambivalence:* ambivalence about the HPV vaccine for men; (3) *altruism*: considering men’s roles and responsibilities in HPV transmission; and (4) *trusted sources*: identifying trusted sources of information. We discuss these themes below, drawing similarities and differences from the cross-cultural analysis. Findings are considered against our study aim and the implications that they have for communication, given the current problem of suboptimal uptake of the HPV vaccine amongst men. Illustrative quotes are provided along with codes (country/focus group number/participant number).

## The ‘Girl Vaccine’

The HPV vaccine was seen as a ‘girl’ vaccine from men’s accounts. Most Scottish and Spanish young men had little knowledge or understanding of the HPV virus or HPV vaccine at the time of the study. However, some participants associated the HPV vaccine exclusively with girls’/women’s health. For example, from this Spanish focus group:

*I: What do you know about the HPV virus…and vaccine?*

*P3: I never heard anything.*

*P6: I think it only affects women, right? [overlapping] from what I have read*

*I: Mostly or only?*

*P6: I think only women [Spain/FG1]*

Clearly, there were tentative associations made with women and the HPV virus and vaccine as seen in: “I think it only affects women, right? ” (Spain/FG1/P6). In addition, girls/women were seen to be the holders of information about the vaccine and distinctly contrasted with men’s lack of knowledge and information. For example:

*P3: Men don’t know that this vaccine exists apart from the fact that I think the government should subsidise it. I didn’t know about the vaccine before coming here.*

*I: That is also important.*

*P3: Women know all this information since they were 13.*

*I: Right when they are 12.*

*P3: But they do know the information I think. [Spain, FG3]*

Above, young men positioned themselves at the periphery of information and knowledge for the HPV virus and vaccine compared with women who apparently knew this information from an early age. This emphasis on being at the margins of public health messaging was in keeping with current vaccine implementation policies for the HPV vaccine in these countries at the time of the study (single-gendered vaccine programmes). Many were surprised that the HPV virus could affect men’s health at all prior to the focus groups, and men appealed to their governments to subsidise the HPV vaccine for boys/young men as well as girls, given the prohibitive costs with obtaining the vaccine privately (as seen above).

Some young men, however, had actively searched for information prior to their attendance at the focus groups. Others had recollections of the HPV vaccine being delivered at school for girls in Scotland, once prompted with stimulus material during the focus groups to elicit discussion (local information sheets that were available in each country for girls or in the US boys/young men):

*I: Anybody else know anything about the [HPV] virus?*

*P3: I didn’t have any recall of it at all but reading the thing about being offered to young girls in Scotland, I do remember being at school and it being offered to all the girls. Obviously the boys didn’t have to bother with it at all, because it was seen as nothing to do with us at all so I didn’t really know anything [about] it at the time. Yes, this is about the extent of what I know about it now […] Just all the girls in my class that would all go down to the assembly hall to get their vaccine while the guys just sat in class and didn’t bother going. I did kind of know it was related to cervical cancer but that was just from what I’d heard people saying about it. [Scotland, FG1]*

Above, the physical separation of girls from boys during school assemblies to receive the HPV vaccine in the Scottish context is poignant as it emphasises the further psychological separation of boys from the messaging of this vaccine at the time of the single-gendered programme. Here, the HPV vaccine is perceived as something that ‘the boys didn’t have to bother with it all’ and having ‘nothing to do with us at all’. The participant recalls the links with cervical cancer for women that he gleaned indirectly through ‘just what I’d heard people saying about it’. Here, the HPV vaccine was again relegated exclusively to women’s health and body. In sum, men were psychologically separate from the HPV virus and vaccine due to anatomy, physically separated during school assemblies, and socially separated from information and knowledge about the virus and vaccine.

In the US where the HPV vaccine was already available for boys/young men as well as girls/young women, participants indicated a range of awareness of the vaccine. However, despite the HPV vaccine being available for boys/young men, some were unaware of the health benefits for young men and that men could be directly affected by the HPV virus at all; this resembles our earlier Spanish and Scottish extracts. One young man from the US talked about how his eventual decision to be vaccinated with the HPV vaccine arose after a routine health visit for his sister:

*Well, the reason that I heard about the HPV vaccine is because my sister went in to the doctor's, and she was told, there's this new vaccine out, you should probably get it, it's for HPV. And then during the discussion it came out that it was also for men, so I got it as well. So that's how I heard about it. [US/P1/FG 6]*

Unlike the previous Spanish and Scottish contexts, in this US context the HPV vaccine was seen as relevant for men (in keeping with the gender-neutral vaccine programme there). However, what is significant is that it was through the sister’s routine health visit with her physician that P1 learned about the availability of the HPV vaccine for men. It is interesting that P1 learns about the vaccine not through direct physician recommendation for his own health, but rather through indirect sources and a female health encounter. This negotiates a further gendered dimension where women remained not only the chief recipients of the HPV vaccine but also the potential transmitters of HPV knowledge and information, like in our Scottish and Spanish data.

## Vaccine Ambivalence

Once young men had read and looked at information sources about HPV and the HPV vaccine which were provided as stimulus prompts for discussions in focus groups, they considered their own health risks in relative terms to girls/young women and evaluated these as being considerably less important. Women were seen as ‘higher risk’ for the HPV virus. When it came to the logic of the rationing of resources in single-gendered contexts, young men mostly considered that those at ‘higher risk’ from the virus should understandably be considered higher priority although equality arguments from the Scottish and Spanish discussions were also apparent. Participants’ perceptions of ‘high risk’ groups were identified from the discussions to include girls/women; men-who-have-sex-with men (MSM); and promiscuous individuals. For example:

*P2: The risk is higher for [women]… I have read somewhere 1% [risk to men], I don’t know. In my opinion it’s a very small percentage compared to women, then … on the one hand, if the virus also causes health problems in men they should get the vaccine, why not? They are also human beings. On the other hand, from an economic perspective I think the best thing [is] to give all women the vaccine since they have more possibilities of getting the illness than men.*

*P3: But even though they [men] have less possibilities they shouldn’t have fewer opportunities.*

*                 P2: Man! From an economic perspective… sorry*, *sorry. [Spain, FG3]*

Clearly the rationing of scarce resources was debated between an economic versus an equality argument which follows public health debates. The idea that women should be the prime recipients of the HPV vaccine due to higher health risks appeared logical against the notion that men should also have access to the vaccine given the health benefits. It was apparent throughout that ‘othering’ or distancing of self through minimizing their own ‘low’ personal risks through reference to other ‘high risk’ categories (through gender, sexuality or behaviour) worked to further distance the young men from the need for the vaccine if they did not fall into those ‘high risk’ categories.

In the US context where the HPV vaccine was available to young men, a similar sense of ‘othering’ or distance took place despite the vaccine availability. In the following account, P1 emphasised his ambivalence towards the HPV vaccine despite knowing the vaccine was available for men:

*I think I also heard about it for the first time from my doctor…it was presented as a very kind of optional, “you can do this but it's not crucial to your health”.... And without a strong push from the doctor, I was kind of, you know, ambivalent about whether I got it or not. It wasn't enough to push me to do it. But you know, I think it would be different if I was female. [US/FG 6/P1]*

Above, two US young men were discussing their experiences with learning about the HPV vaccine directly from their doctors and their different decision-making. In both instances, the HPV vaccine was presented as something that was seen as ‘optional’ but not necessarily crucial to men’s health by their doctors. Both emphasised the lack of direct recommendation for the vaccine from the doctor with the message that this was not crucial or ‘doesn’t affect you much’. Without a strong recommendation or ‘push’ from their doctors, young men reported ambivalence about the HPV vaccine and decision-making. P1 employed ‘othering’ to distance himself from the relative risks for his own health and his decision not to get the vaccine in comparison to the high-risk group (women). Some worries about vaccine side effects and the novelty of the vaccine for men also emerged in US accounts along with concerns about long-term effects around the US being a ‘pilot’ country for offering the vaccine to men, unlike in the Scottish and Spanish contexts.

## Altruism

Across the corpus data young men negotiated their own roles and responsibilities for sexual health and the HPV virus. What was apparent was that there were similar accounts where young men aligned themselves with responsible and morally conscious individuals within the broader context of relationships. For example:

*If you have a wife and you are going to get married, well you are not going to do this to your wife! [infect her with HPV virus][Spain/FG2/P6]*

*I think for men it’s not really a question of their own health it’s more a question of the health of others that they’re going to be sexually active with. [Scotland/FG4/P2]*

*I just want to go off on the benefit directly to me…there was the element of just with any sexually transmittable disease, my partner’s safety and the direct benefit to any partner I may have, be it my girlfriend, be it someone I just met, what have you… if I have it [it] won’t necessarily adversely affect me but I would feel some sort of ethical and moral standing then if I passed it on to someone else. [US/FG1/P1]*

Above, young men similarly considered their relationships and responsibilities to ensure that they did not transmit infections to sexual and romantic partners. What is apparent is that responsibilities were seen as less directly related to men’s own health benefits (‘it won’t necessarily adversely affect me’) but rather the indirect benefits for their partners and broader ‘ethical and moral’ aspects of avoiding transmission of sexually transmitted infections within the context of relationships. In this way, young men appeared as protectors of their relationships (young women and/or romantic partners which were not specified as same-sex or opposite-sex in the discussions) drawing on constructions of masculinity as protectors.

## Trusted Sources

Although stimulus materials were provided to young men in the focus groups given that they indicated little/no knowledge of the topic matter, we considered the main sources of information that young men had sourced themselves regarding information and knowledge for the HPV virus and vaccine and which sources that they perceived to be trustworthy. Across the corpus data, health professionals were seen as the main sources of trusted information regarding HPV and the vaccine. Health and medical websites and authorities were regarded as trusted sources of information for HPV, which were evaluated critically. However, despite the significance of these trusted sources, within the context of men’s health, opportunities for these health encounters were seen to be limited compared with women. Young men considered that women were required to seek health advice to a greater extent due to reproductive issues (i.e. which meant they were more familiar with help-seeking) whereas young men, by contrast, were seen to be less concerned or familiar with needing to seek help for health matters within constructions of masculinity and the ‘done’ thing. For example:

*Just in my experience, particularly in Scotland, men just don’t bother ever really going to the doctor or whatever. Like, they do look after themselves, they do sports and physical activity and stuff, but in terms of just going to the doctor and seeking out proper healthcare, it’s not really the done thing, if you know what I mean. [Scotland/FG3/P3]*

Unlike our previous analysis of information sources with young women for the HPV vaccine [29, 30], young men did not report peers as a main source of information and knowledge for the HPV vaccine, nor did they refer to media reports (apart from those given in the focus groups as stimulus material). Privacy was another pertinent issue that was highlighted from the US data in terms of the privacy of the yearly physician consultation to discuss their overall health and the HPV virus and vaccine, and they compared this to the large school-based vaccination in Scotland unfavourably. Men in the US reported having the potential to discuss HPV with their physician rather than friends and peers, although they did recognise that this opportunity was not always taken up by peers, which raised similar questions about opportunities and interactions for help-seeking amongst young men. A further dimension to the information source analysis was that young men reported more *indirect* rather than *direct* information sources for the HPV virus and vaccine, and this often appeared through a context of female health. For example, as seen in the above themes surrounding school-based programmes or medical consultations for female health. Overall, girls/women appeared to be the main recipients of the HPV vaccine in all contexts and carried the main burden for vaccination and information/knowledge.

## Discussion

From our purposive sample of participants in each country, we noted that more US participants indicated that they had heard of the HPV virus, the HPV vaccine, knew that HPV was linked with cancer, and agreed that boys/young men should be vaccinated with HPV compared with the Scottish and Spanish sample. Only some US participants reported that they had been previously vaccinated with at least one dose of the HPV vaccine 6/16 (38%). This is in keeping with the US gender-neutral vaccine policy through private provision at the time. Finally, we note that the 38% of the US sample who reported being previously vaccinated with at least one dose of the HPV vaccine resembles the wider national average reported in the US, giving confidence in our sample characteristics [20].

Given that suboptimal HPV vaccine uptake rates persist amongst boys/young men despite efforts to include boys/young men in vaccine programmes [15, 19–21], this qualitative cross-cultural analysis amongst young men’s in-depth accounts about the HPV vaccine provided an opportunity to give insights into men’s HPV communication needs at different vaccine policy stages from single and gender-neutral contexts. There has been an increase in countries which now offer gender-neutral vaccination campaigns as opposed to single-gendered HPV vaccine programmes which are recommended [2–5]. Despite our data being originally collected in 2015, this cross-cultural analysis allowed unique consideration of some important behavioural insights and considerations according to different HPV vaccine policies and implementation which are continually changing and particularly given the mixed messages around HPV vaccination for boys/young men and its importance [22]. Spain and Scotland now offer gender-neutral vaccine programmes like the US and many other contexts. Overall, our cross-cultural analysis highlighted overarching uncertainties for the HPV vaccine amongst young men regardless of the availability of the vaccine in different contexts, which is significant and which we discuss below.

Our finding that our young men navigated the HPV vaccine as largely a ‘girl’ vaccine is not surprising given that Scotland and Spain followed a single-gendered vaccine policy at the time and is in keeping with literature in other contexts [18, 36]. However, the legacy of the ‘girl’ vaccine is important to consider when moving from single to gender-neutral contexts and communication about the HPV vaccine. The feminisation of the HPV vaccine has been previously described in the US context, with the intersection of science, politics, economics, and gender norms affecting implementation, gender inequality, and lower HPV vaccine uptake rates there [37]. Similarly, young men in the Scottish and Spanish contexts held very limited knowledge and understanding of the HPV vaccine and relegated this to girls, which is in keeping with previous and current research [18]. For example, a recent qualitative study with parents and adolescents in Andalusia, Spain, showed poor knowledge of HPV and the vaccine overall and limited discussions in families about this topic [38]. In the US context, however, while more young men had indicated that they had heard about the HPV virus and vaccine compared with our Scottish and Spanish participants, and while more US men there reported being vaccinated with at least one dose of HPV (38% compared with the national average of 34%), not all of our US participants appeared knowledgeable and informed in this context either and points to the overall limited knowledge reported in US contexts despite being from a gender-neutral vaccine country [15, 18].

Tied to the above, our finding about young men’s ambivalence about the HPV vaccine in US contexts is noteworthy. From our analysis, young men similarly worked to position themselves at the periphery of the HPV vaccine. They did this in the following ways: some young men appeared to be unaware that the HPV vaccine could benefit men’s health at all; others talked through how they came to be aware of the HPV vaccine through personal health encounters or mediated through a context of female health (mothers, sisters’ health and awareness). Hence, despite HPV vaccine availability in the US, albeit through private provision and insurance, young men appeared ambivalent and unclear about the benefits and implications of this vaccine. Cost was indicated in this study in the Spanish and US contexts and has also previously been indicated in the literature to be a barrier towards vaccination [15, 39]. Further, our young men’s considerations of who was at the highest risks for HPV were apparent during the focus group discussions across the cross-cultural corpus and may have worked to distance themselves from the relevance and importance of the vaccine through reference to ‘other’ high-risk groups including women for cervical cancer, MSM, and promiscuous individuals. Underestimating men’s HPV risks has been reported in North American contexts elsewhere [40]. Despite the different cultural contexts then, young men appeared largely at the periphery of the HPV vaccine in terms of disease aetiology, knowledge and information, and vaccine provision. By contrast, young women were positioned to have the greatest cancer burden, and the higher risk burden for receiving and distilling HPV information and knowledge and for personal vaccination behaviour [36].

Our finding that when young men considered their own roles and responsibilities in HPV transmission, they resorted to a moral and responsible discourse is encouraging and has parallels with encouraging altruistic behaviours and personal health benefits found in a US setting [41]. While young men appeared unclear overall about the importance of the HPV vaccine for their own personal health, they considered that it was important to protect others within sexual and romantic relationships, drawing on constructions of masculinity of men as protectors of their romantic and sexual relationships as well as ‘herd’ immunity. In keeping with consistent literature, health providers, authorities, and medical websites were regarded as trustworthy sources of information for the HPV vaccine according to our young men participants [40, 42]. What is also noteworthy is young men’s reported health encounters/help-seeking and constructions of masculinity that minimised such encounters. Khalil et al.’s [21] finding that young men in the US had low HPV uptake rates despite their yearly physician appointment is significant and, in our study, only 38% of our US sample reported being vaccinated with at least one dose of the HPV vaccine. Men appeared uninformed and ambivalent about the HPV vaccine there. It is important to maximise vaccination information in health encounters in specific contexts including schools and health provider encounters [21, 40]. Vaccine worries were mentioned in our study amongst the US participants, but this was not apparent to a large extent, which contrasts with findings about greater vaccine worries amongst young women indicating possible gender differences [29, 30, 35]. Gender differences for other vaccine intentions have been previously reported, for example, the COVID-19 vaccine [43]. Such differences could be further explored, or they may have been due to the implementation stages of the vaccine programmes for boys/young men.

## Limitations and Further Research

This cross-cultural analysis from qualitative studies in Scotland, Spain, and the US is based on data originally collected in 2015, which we acknowledge is relevant to that time period. However, it offers a unique opportunity to understand behavioural insights about the persistent problem of suboptimal HPV vaccination amongst young men in countries with different, changing implementation programmes, and such insights are relevant to contexts undergoing changes from single to gender-neutral vaccine programmes. There are key learnings which are important from changes to vaccine policy, which we have attempted to draw out. Changes to vaccine policy to include boys in the Scottish and Spanish contexts are in keeping with Europe’s Beating Cancer Plan [13]. Historically, the implementation of the HPV vaccine for boys coincided with the COVID-19 pandemic [2] which affected HPV vaccination implementation efforts for boys alongside WHO recommendations to temporarily suspend this vaccine for boys [22]. Efforts were made to follow rigorous procedures in the study, including the data collection and analysis. The study relied on volunteers from university and higher education colleges and thus may not be generalisable to all young men in these contexts. Our cross-cultural analysis of accounts from young men from different countries (Scotland, Spain, and the United States) highlighted that despite living in different countries which had different vaccine policies at the time (single versus gender-neutral vaccine polices) that similar accounts were produced around the uncertainties for HPV vaccination for men. We consider this to be a significant finding, which has implications for public health communication in these and other similar cultural contexts. We note that the accounts produced may be similar due to being from Western, industrialised nations. Other accounts may be possible in other countries. We make no claims about replicability. However, the findings here may be transferable to other cultural contexts with similar cultural norms and vaccine histories and can be explored in future studies. Future research should verify these findings over time as vaccine policy and implementation changes, with other non-university/education college samples, including vulnerable and marginalised young men, such as those with disabilities or considered ‘high-risk’, such as MSM [44].

## Recommendations

Public health authorities and HPV vaccine campaigns in different contexts should make use of both *direct and indirect* appeals to boys/young men for HPV vaccination. Specific and targeted public health messages should directly address the health benefits of the HPV vaccine for boys/young men for men’s cancer prevention and genital warts. What is significant is that even in contexts where the HPV vaccine was offered, there was poor knowledge about the direct health benefits for men compared with women. Countries seeking to implement the HPV vaccine for gender-neutral programmes and those at early stages of implementation (such as the UK and many European countries) should be cognisant of these aspects. The HPV vaccine as a cancer-prevention vaccine for oral, penile, and anal cancers, and not purely cervical cancer, should be highlighted along with the prevention of genital warts as this may be effective in appealing to parents and to boys/young men. For example, focusing on communicating messages about cancer prevention for penile, anal, and oral cancers was found to be more effective than focusing purely on genital warts alone [45]. Indirectly, the finding that young men reverted to the construction of the responsible protector within sexual and romantic health relationships who wished to avoid the undesirable stigma of transmitting sexual infections is also important. Public health messages could frame and tailor the relationship aspects in the cases of young people embarking on romantic relationships within broader sexual health education to promote the protection of their own and their partner’s sexual health, such as for condom use, for example, alongside compatible messaging about herd immunity [40, 46]. The finding that even in the US context where the vaccine was available to boys/young men at the time of the study, young men’s health appeared regulated via a context of female health suggests that informing women/girls of the benefits for both genders may also be effective in reaching boys/young men. However, clearly, the burden for information transmission and vaccination should not rely on women solely given the gendered notions of this vaccine, and gender-neutral vaccine campaigns that focus on the population at large are desirable. Vaccine ‘novelty’ for the HPV vaccine amongst young men should also be borne in mind when delivering health messages as these can affect vaccine hesitancy, and this may be particularly present given the COVID-19 vaccine. Clear messages about the safety of the HPV vaccine in boys/young men should be communicated from trusted health sources and providers. The finding that health providers and medical/health websites were still the most trusted source of information means that direct recommendations from health providers in specific countries are important and opportunities for such encounters should be encouraged to promote uptake amongst boys/men. To achieve gender-neutral vaccine programmes and promote the vaccine amongst boys/young men, health authorities in different contexts need to be proactive and emphasise the health benefits and safety of the HPV vaccine directly for boys/young men and for the wider population.

## Conclusion

Through close, in-depth analysis of young men’s communication needs for the HPV vaccine in this cross-cultural analysis, we highlighted how young men were physically, psychologically, and socially separated for the HPV vaccine in these contexts. Physically, HPV vaccination appeared in spaces which excluded boys/young men (including school assemblies, and private health encounters with doctors for example). Psychologically, young men distanced themselves and minimised their risks for the HPV virus which they contrasted with other higher ‘risk’ groups including women, MSM, and promiscuous individuals. Socially, knowledge and information provision appeared to exclude young men as they were not the main targets or received mixed messaging for the HPV vaccine within current public health campaigns in their countries, including the US. With widening emphasis on gender-neutral vaccine programmes and a wider context of vaccine hesitancy in contexts such as Europe, it is important that public health communication and education highlight direct health benefits for boys/men’s health including cancer prevention for oral, anal, and penile cancers as well as genital warts. It should also incorporate messaging about the indirect benefits on relationships and the importance of increasing ‘herd’ immunity efforts for direct benefits for women through cervical cancer prevention when in heterosexual relationships and emphasise men’s roles in prevention [42, 43]. Renewed communication efforts that are tailored to specific cultural contexts are particularly salient given the mixed messaging about the importance of boys/young men HPV vaccination implementation from professional bodies and the effects of COVID-19 on vaccine hesitancy.

## Data Availability

Aggregated data-sets may be available from the corresponding author. Individual data-sets require intitutional review board.
